# Clinical Features and Follow-Up of the First Two Cases of Mpox From Trinidad and Tobago

**DOI:** 10.7759/cureus.53149

**Published:** 2024-01-29

**Authors:** Robert Jeffrey Edwards, Jonathan Edwards, Gregory G Boyce

**Affiliations:** 1 Human Immunodeficiency Virus (HIV), Medical Research Foundation of Trinidad and Tobago, Port of Spain, TTO

**Keywords:** trinidad, stigma, genital lesions, msm, mpox

## Abstract

Mpox is a viral zoonotic disease that is endemic in Central and West Africa and belongs to the *Orthopoxvirus* genus. A global outbreak of mpox began in May 2022, mainly due to the transmission of the clade 11b virus through person-to-person contact with the lesions or scabs of a person infected with mpox. The data on mpox infection in the Caribbean is sparse. Here we report the clinical features and follow-up of the first two confirmed cases of mpox in Trinidad and Tobago (T&T). Both patients were men who have sex with men (MSM) who presented with genital lesions and expressed concern about increased stigma towards their already marginalized community.

## Introduction

Mpox is a viral zoonotic disease that initially occurred in tropical rainforest areas of Central and West Africa and is a member of the *Orthopoxvirus *genus [1]. There are two clades of the virus: clade 1 is the Congo Basin/Central African strain, which is correlated with more severe disease, and clade 11 is the West African strain, which is associated with less severe disease [1]. Mpox is transmitted to humans through direct or indirect contact with lesion material or body fluids (e.g., respiratory droplets) from an infectious person, contact with contaminated fomites, or contact with infected animals [2, 3]. In May 2022, several countries with no previous cases of mpox reported sustained community spread of the disease among persons with no recent travel history, primarily driven by person-to-person contact among men who have sex with men (MSM) [4]. The Republic of Trinidad and Tobago (T&T) comprises a single nation and is the southmost island of the Caribbean chain, with a population of approximately 1,405,646 people (2022 mid-year estimate). Due to the convergence of the HIV pandemic and the outbreak of mpox which both disproportionately affect MSM, this has resulted in enhanced awareness among healthcare professionals and members of the general population; thus, care must be taken to reduce stigma and discrimination against members of this key population group.

## Case presentation

Case one

A 53-year-old MSM was seen on July 8, 2023, as he was worried about being exposed to a sexually transmitted infection (STI). The patient was asymptomatic and said his partner was recently treated for gonorrhea and chlamydia by his general practitioner, so the patient was screened for gonorrhea, chlamydia, syphilis, and HIV and was given empiric treatment (a single dose of 500 mg of intramuscular ceftriaxone to treat gonorrhea and oral doxycycline 100 mg twice daily for seven days to treat chlamydia). The patient’s partner, AA, did not have an appointment but asked for medication to ease his severe perianal pains. On examination, there were shallow perianal ulcers, so he was treated empirically for genital herpes simplex with sitz baths and acyclovir 400 mg three times daily for seven days. He was asked to return for an investigation and further management in two days.

On the next day (July 9, 2023), patient one sent via WhatsApp a photo of an asymptomatic umbilicated lesion that he said developed on his penis. Suspecting mpox, the patient was asked to come in on July 11, 2023, so that swabs and viral transport media could be procured. In addition, the patient was told to ask any of his recent sexual partners if they had genital or anal lesions or lesions on the hands and feet. The patient later called and said he spoke with his general practitioner, who expressed skepticism that mpox can be diagnosed from a single asymptomatic genital ulcer.

Patient one had recent sexual contact with four MSM individuals. Two (AA and BB) of these were his recent regular partners, and the other two (CC and DD) were a couple with whom they all had sexual relations. Of the four contacts, three had skin or anogenital lesions, and one had flu-like symptoms but no skin lesions. None had a recent travel history; however, all five people had sexual contact with a person (MSM) who had recently returned from Mexico, two days after they arrived in T&T. This person claimed he was vaccinated for mpox in the USA in July 2022 and that he was asymptomatic.

**Figure 1:**
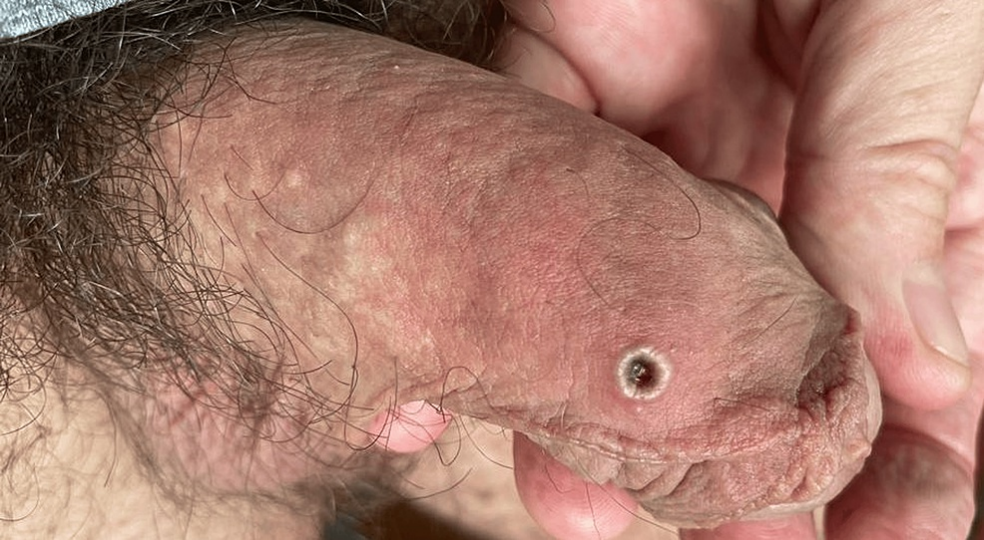
Umbilicated lesion on the penis

On examination, patient one had an umbilicated penile lesion (Figure 1) and tender right inguinal lymphadenopathy.

A swab was taken from the umbilicated penile lesion and immediately transported in viral transport media to the Caribbean Public Health Agency (CARPHA) for polymerase chain reaction (PCR) analysis.

**Figure 2:**
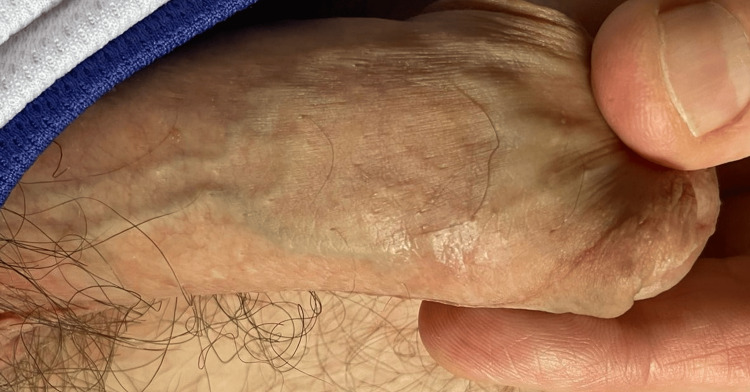
Lesion healed with a small depressed scar after 21 days

Patient one tested positive for mpox (the West African strain) and was asked for permission to give contact information to the Ministry of Health for contact tracing to be conducted. After a lot of persuasion, patient one reluctantly agreed, fearing stigma and discrimination because he is MSM, he was the first case of mpox in T&T, and there was no treatment for mpox in T&T. The patient’s contact information was given to the County Medical Officer of Health (CMOH), who advised the patient to go into isolation (for 21 days), and contact tracing was initiated. The entire group of five people went into isolation (they rented a property in the country); none of the others underwent mpox testing, as they assumed that they would all test positive. Patient one gave daily updates and photos of how the condition was progressing. Three days after the appearance of the penile lesion, he complained of headaches, fatigue, malaise, and chills (which lasted for two days and then resolved). The venereal disease research laboratory (VDRL) was non-reactive, the HIV test was negative, and the nucleic acid amplification tests for chlamydia and gonorrhea were negative. The patient was treated supportively, and the lesion on the penis healed after 21 days with a small depressed scar (Figure 2).

Case two

Patient two, a 26-year-old MSM, was referred from the STI clinic on July 11, 2023, for evaluation of a “strange” rash. The patient had a history of fever and body pains for two days beginning July 2, 2023, which resolved and was followed two days later by a skin rash that began in the perianal region and then spread to the penis, trunk, and fingers. The rash in the perianal area was quite painful. The patient had no recent history of travel.

**Figure 3:**
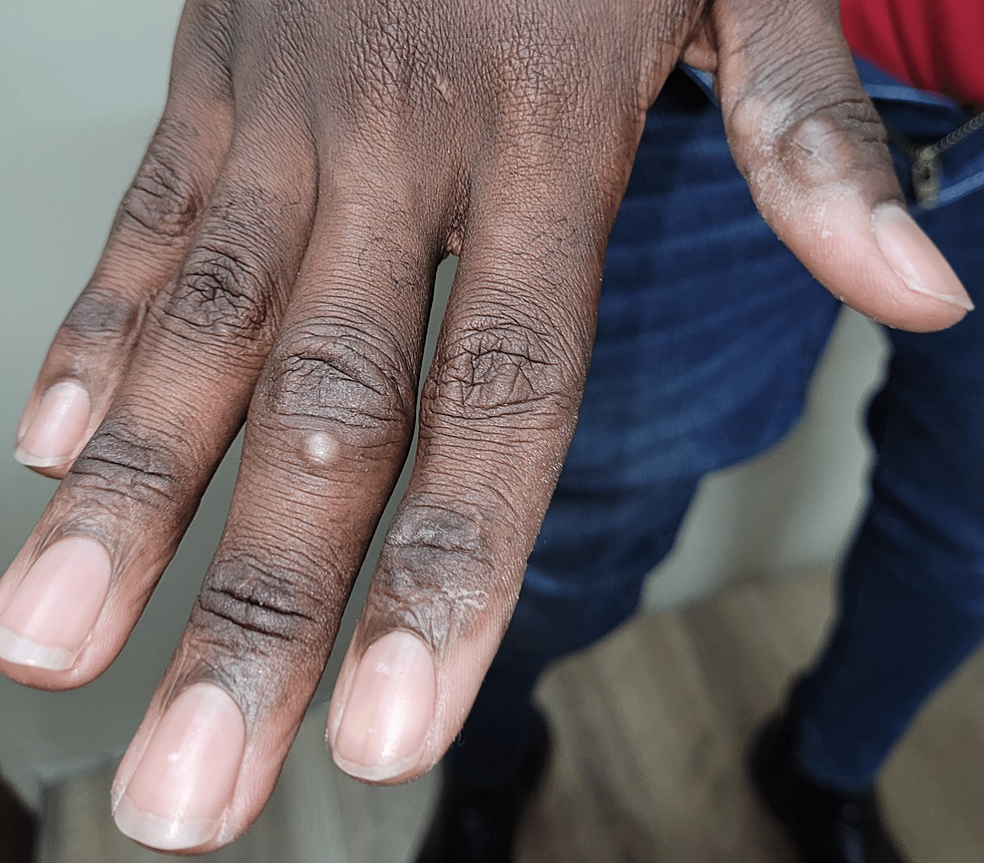
Two vesicopustular lesions on the medial aspect of the right thumb and a pustular lesion on the dorsum of the third right finger.

On examination, patient two had two vesicopustular lesions on the medial aspect of the right thumb and a pustular lesion on the dorsal aspect of the third right finger (Figure 3).

**Figure 4:**
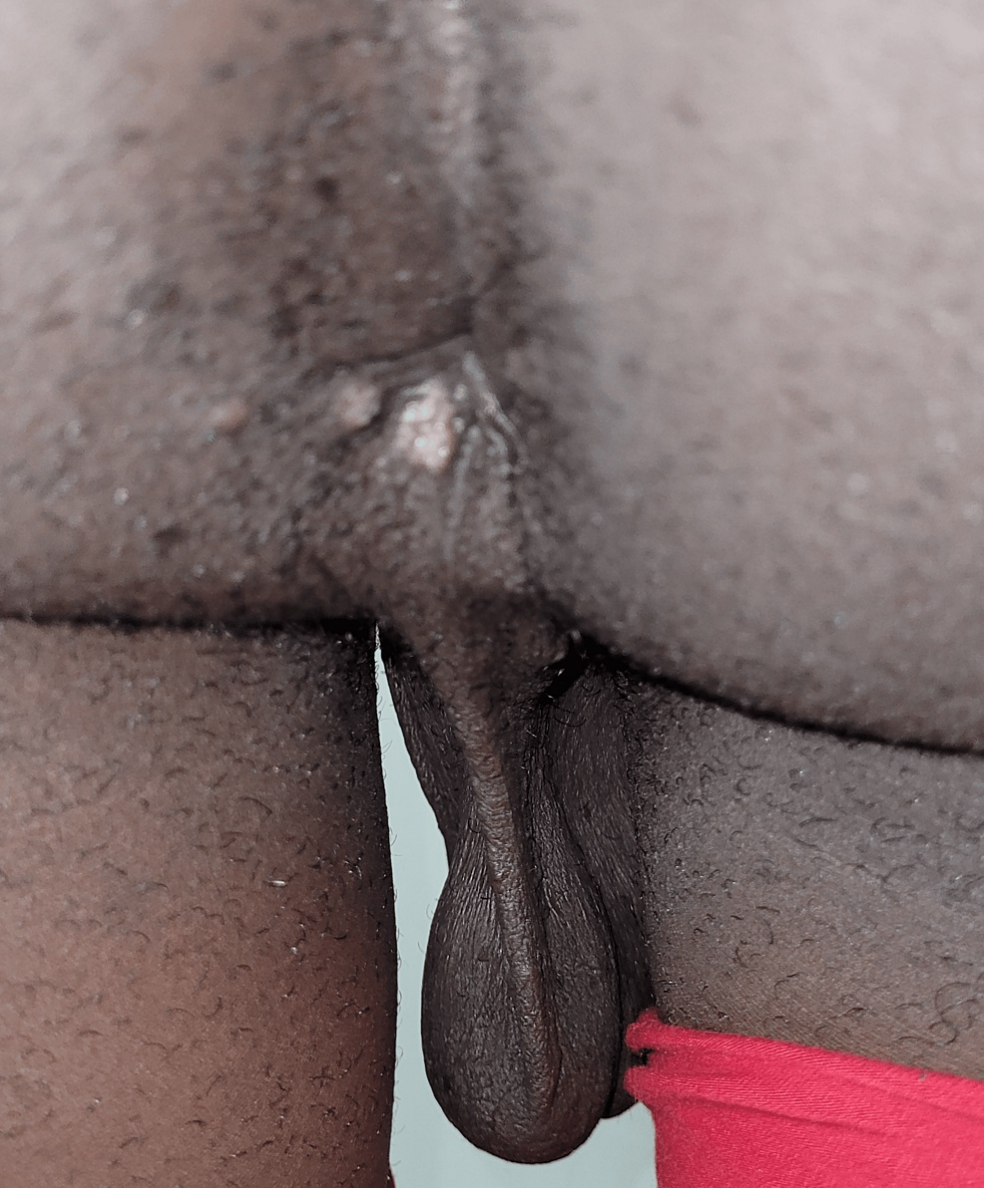
Perianal ulcers found in patient two

The patient had two shallow penile ulcers and a few perianal ulcers (Figure 4).

**Figure 5:**
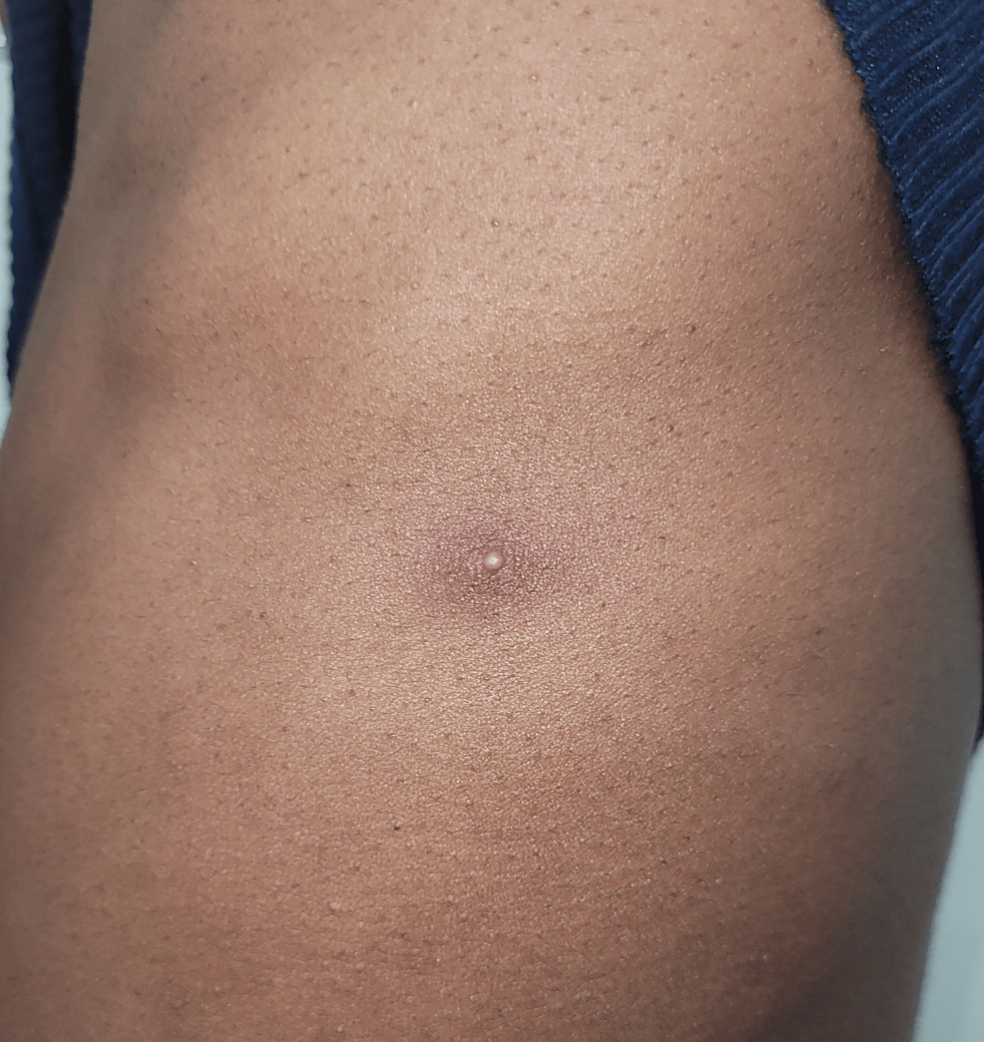
A single pustular lesion with surrounding erythema on the trunk

In addition, the patient had a single pustular lesion with surrounding erythema on the left trunk (Figure 5).

He also had inguinal and axillary lymphadenopathy.

Patient two had one MSM partner with whom he had recent sexual contact. This partner was HIV-infected (on antiretroviral therapy) and was asymptomatic. The patient indicated that there were five people with whom he had “close physical contact” over the past four weeks, and all were asymptomatic. Swabs were taken from the perianal lesions and the pustular lesion on the trunk and immediately transported in viral transport media to CARPHA for PCR analysis. Blood samples were sent for HIV and syphilis (VDRL) testing.

**Figure 6:**
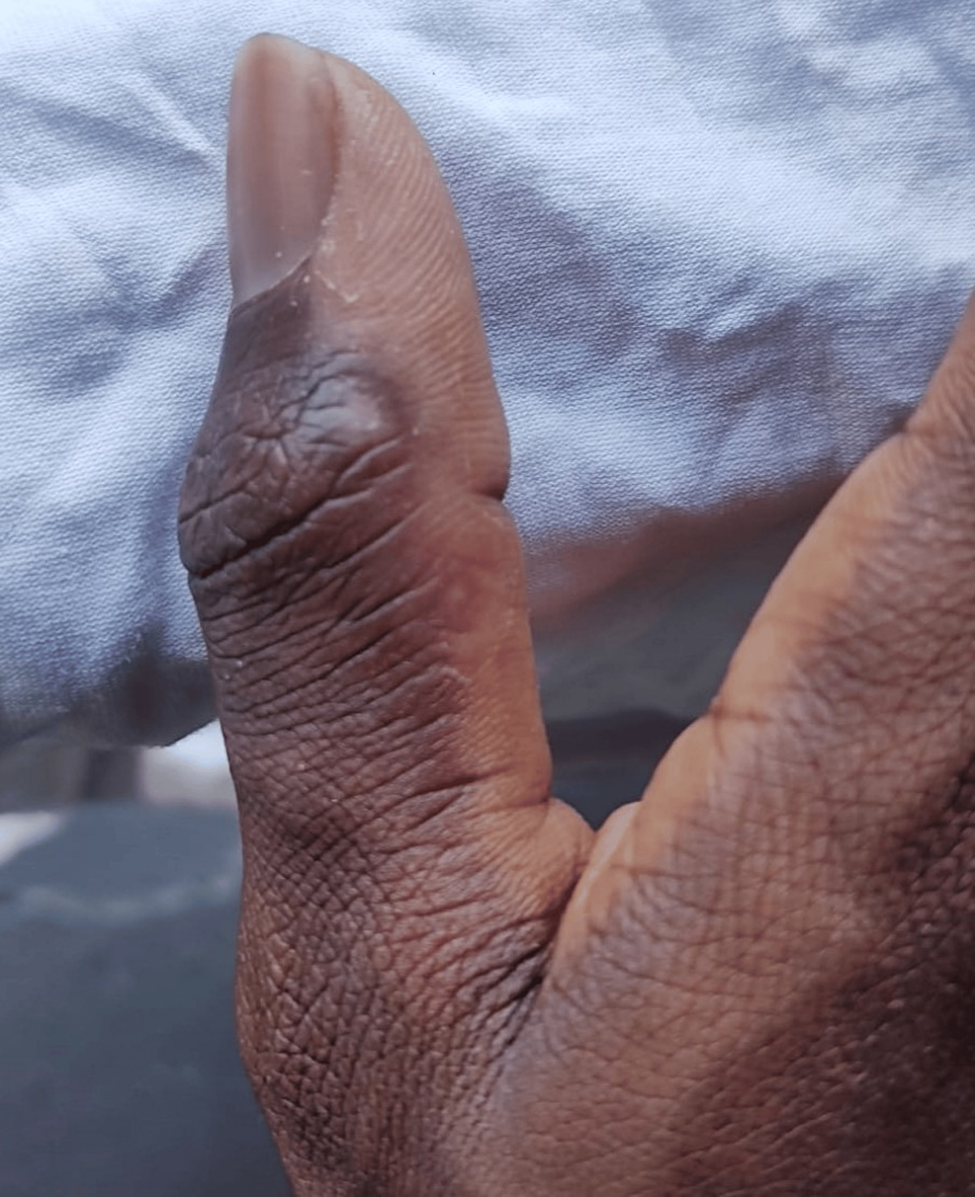
Healed lesions on the thumb covered by new skin after 27 days

The patient tested positive for mpox (the West African strain) and was asked for permission to give his contact information to the Ministry of Health for contact tracing to be conducted. He readily agreed, but he expressed concern about the potential stigma associated with him being MSM. He was given an isolation letter for 21 days, and a few of his asymptomatic contacts received the Jynneos vaccine, as T&T had a stockpile of 2,300 vaccines. The patient was treated conservatively and gave daily updates and photos of how the condition was progressing. The anogenital lesions healed totally after 16 days, and the lesions on the fingers were totally healed after 27 days (Figure 6).

The HIV test was negative, and the VDRL was non-reactive.

## Discussion

Laurenson-Schafer et al. [4] conducted a systematic analysis of the global surveillance data of 82,807 cases of mpox and found that cases were primarily due to the mpox clade 11b virus, and the most common clinical features were any rash (82.6%), fever (59.6%), skin/mucosal lesions (excluding oral/genital lesions) [52.2%], anogenital lesions (46.4%), and headache (32.8%) [4]. Patients who were immunosuppressed were more likely to develop severe mpox infections characterized by larger, more extensive lesions and severe bacterial infections involving the skin, hematologic, and respiratory systems [1], and deaths have been reported in patients with advanced HIV infection [5]. Nunez et al. [6] conducted an observational study of the clinical features of laboratory-confirmed mpox in Mexico and found that being MSM, anogenital lesions, inguinal lymphadenopathy, pustules, and HIV infection were associated with a confirmed diagnosis of mpox [6]. In our study, both patients were MSM who presented with anogenital lesions and inguinal lymphadenopathy, and patient two had pustules on the fingers and trunk.

Differential diagnoses of mpox infection include syphilis, herpes simplex and other STIs, varicella, bacterial skin infections, scabies, bullous skin diseases, and medication-associated rashes. A laboratory diagnosis of mpox is confirmed by testing the skin/lesion material using PCR for the orthopoxvirus. The disease is usually self-limited and generally resolves in two to four weeks, and the patient is no longer infectious when all the scabs fall off and a new layer of skin has formed underneath [1]. Treatment is mainly supportive and involves the care of the rash by washing the hands before and after touching the lesions, keeping the lesions covered as much as possible, hydration, and pain relief. There are no specific treatments for mpox infection; however, antivirals used to treat smallpox (tecovirimat) have been shown to be beneficial in patients with more severe diseases [7]. Vaccines are approved for the prevention of mpox and are recommended for persons at risk, such as close contacts of patients with mpox as post-exposure prophylaxis or persons who belong to high-risk groups (e.g., MSM) for mpox infection. In healthcare institutions, training and reinforcement of good public health practices will aid in prevention, such as the covering of infectious lesions, wearing a well-fitting mask, hand hygiene, and healthcare workers using appropriate personal protective equipment (PPE) [1].

Both cases were HIV-negative and resolved with supportive care; none were hospitalized. Patient one had four sexual partners with symptoms and signs suggestive of a possible mpox infection, but these persons never underwent testing, and all five persons went into isolation for 21 days. The source of the outbreak of mpox in T&T was never established, as neither of the confirmed cases had any recent travel history nor were the cases epidemiologically linked to one another. Public health measures reduced the spread of mpox in T&T and included isolation of infected cases and quarantine of symptomatic contacts. The Jynneous vaccine was offered to asymptomatic contacts as postexposure prophylaxis, and the vaccine was also offered to healthcare workers who came into contact with these patients. In July 2023, T&T reported four cases of mpox, and there have been no cases since. Patient one's general practitioner was skeptical of a diagnosis of mpox in a patient with a single genital lesion. The STI clinic physician was puzzled that a patient presented with anogenital lesions and pustules on the skin, and one of the authors (RJE) missed a possible case of mpox in patient one's partner AA with severely painful perianal lesions. Thus, physicians should have a high index of suspicion and consider the diagnosis of mpox if the patient is an MSM who presents with anogenital lesions, pustules, and inguinal lymphadenopathy [6].

Due to the convergence of the HIV pandemic and the outbreak of mpox, which both disproportionately affect MSM, both patients expressed concern about possible stigma and discrimination due to heightened awareness among healthcare workers and members of the general population who may perceive mpox as a “gay disease” and may ascribe blame to the MSM community for the spread of the infection [8]. Hence, care must be taken to reduce stigma and discrimination against members of this already stigmatized key population group by promoting harm-reduction, non-stigmatizing messages free from gender and sexual identity bias to members of the general population [9].

## Conclusions

The first two cases of mpox in T&T both presented with genital lesions; thus, physicians should have a high index of suspicion and screen for mpox, especially if the patient is an MSM with anogenital lesions, pustules, and inguinal lymphadenopathy. Both patients were concerned that people in the general population may attribute blame to members of the MSM community for the spread of this disease, so harm-reduction, non-stigmatizing messages should be promoted.
